# Snapping Plantaris Tendon: A Rare Case in a Competitive Dancer

**DOI:** 10.5435/JAAOSGlobal-D-21-00008

**Published:** 2021-05-04

**Authors:** Brady D. Greene, Stacy E. Smith, Jeremy T. Smith

**Affiliations:** From the Foot and Ankle Center, Department of Orthopaedic Surgery, Brigham and Women's Hospital, Harvard Medical School, Boston, MA (Greene and J. T. Smith); the Division of Musculoskeletal Imaging and Intervention, Department of Radiology, Brigham and Women's Hospital, Harvard Medical School, Boston, MA (S. E. Smith); and the STRATUS Center for Simulation in Medical Education, Brigham and Women's Hospital, Harvard Medical School, Boston, MA (S. E. Smith).

## Abstract

Pathology associated with the plantaris includes rupture of the tendon and an association with mid-substance Achilles tendinopathy in some patients. There have only been two previous case reports in the literature in English language describing snapping of the plantaris tendon. We present a case report of a 15-year-old female competitive dancer who described pain and an audible popping at the medial margin of the Achilles tendon while squatting. Physical examination revealed visible and audible popping of the plantaris, and ultrasonography confirmed the diagnosis. After symptoms persisted despite nonsurgical treatment with physical therapy, the patient underwent an open plantaris tenotomy. By 8 weeks after surgery, she had resumed dancing. Twenty-three months after her operation, she reported an excellent outcome and full recovery with no limitations to her physical activity. She reported having no pain, a Foot and Ankle Ability Measure Activities of Daily Living Subscale score of 100, and a Foot and Ankle Ability Measure Sports Subscale score of 100. This case demonstrates a successful course of treatment for this uncommon pathology within the context of a competitive dancer.

Musculoskeletal injuries are frequent among dancers across a variety of genres.^1-3^ In particular, lower extremity injuries may account for upward of 66% to 91% of musculoskeletal injuries among ballet dancers.^4^ Common lower extremity pathologies among dancers include ankle impingement, plantar fasciitis, flexor hallucis longus tenosynovitis, peroneal tendon injuries, lateral ankle ligament injuries, Morton's neuromas, and osseous injuries.^5,6^

The plantaris is a small muscle adjacent to the triceps surae, believed to be involved primarily in proprioception. Plantaris pathology includes tendon rupture and an association with mid-substance Achilles tendinopathy.^7,8^ A paucity of literature exists reporting plantaris pathology among dancers. There have been two previous case reports in the English literature describing snapping of the plantaris: one in a 30-year-old man (nondancer) after a skateboarding injury and another in a 30-year-old male amateur runner.^9,10^ Herein, we describe a third case within the context of a 15-year-old female competitive dancer from whom we have obtained informed consent. We describe her treatment, patient-reported outcome measures, and a brief review of plantaris anatomy, function, and pathology.

## Case Report

A healthy 15-year-old female competitive dancer who participated primarily in ballet presented with a sharp, painful, snapping sensation in her left posterior heel. Her pain had developed over several weeks without a specific traumatic event, change in activity, or shoe-wear. This snapping sensation was located slightly medial to the Achilles tendon mid-substance and occurred when squatting. She reported dancing daily almost year-round and denied previous lower extremity problems, including a history of frequent ankle sprains or recent fluoroquinolone antibiotic exposure.

No clinically notable hindfoot malalignment, asymmetry, swelling, or edema was noted. Left ankle range of motion was 15° dorsiflexion to 40° plantar flexion, and her ankle was stable to anterior drawer testing. She had a normal Thompson test and a negative Silfverskiold test. Focal tenderness was observed at the noninsertional Achilles tendon without swelling. With knee flexion and ankle dorsiflexion (squatting), a visible and palpable snapping was reported just medial to the mid-substance of the Achilles tendon (Supplemental Video 1, http://links.lww.com/JG9/A121). She reported that this maneuver replicated her pain when dancing. The snapping was not replicable with palpation alone.

**Figure 1:**
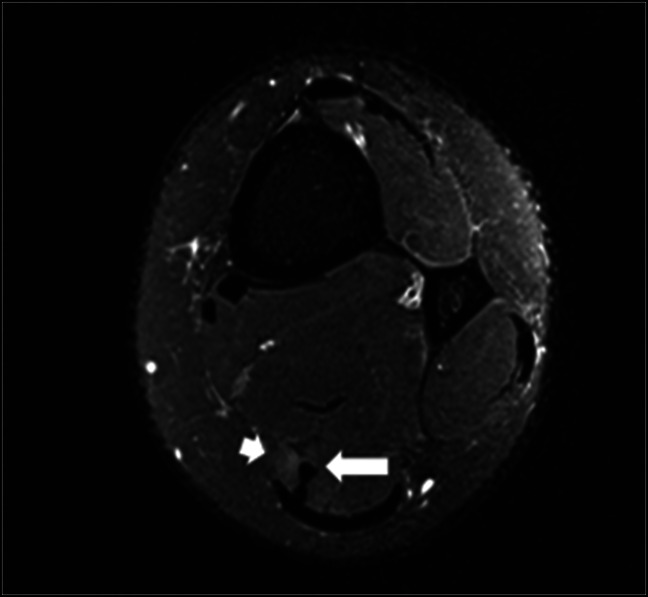
Axial T2FS MR image of the left ankle demonstrating feathery edema within the posterior medial muscle fibers of the Achilles (shorter white arrow) with adjacent anteromedially located low-signal plantaris tendon without tendinopathy (longer white arrow).

Left ankle radiographs demonstrated no acute bony injury, Haglund's lesion, or Achilles insertional calcification. Ankle MRI showed mild feathery edema at the myotendinous junction of the Achilles near the medially located plantaris tendon without evidence of tendinopathy (Figure 1). We initiated treatment with rest from dance and physical therapy, specifically involving gentle stretching, local soft-tissue manipulation, and eccentric training. After 6 weeks, she reported no improvement with persistent pain while squatting.

**Figure 2:**
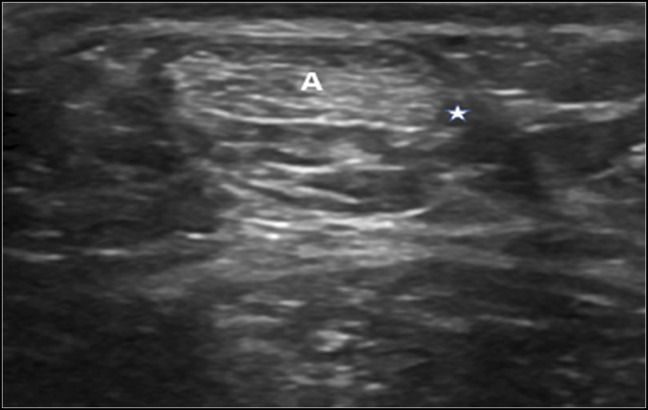
More inferior transverse sonogram demonstrating the anteromedial position of the distal plantaris tendon (*) regarding the Achilles tendon (A).

**Figure 3:**
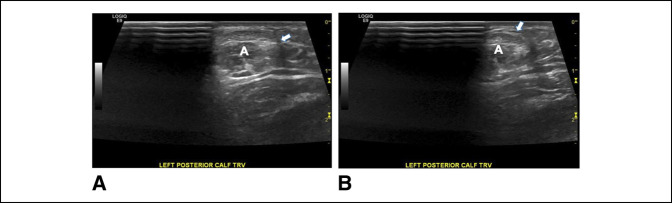
**A**, Transverse sonogram depicting the plantaris tendon (arrow) before subluxation medial to the Achilles tendon. **B**, Transverse sonogram demonstrating subluxation of the plantaris tendon (arrow) regarding the Achilles tendon (**A**).

A dynamic ultrasonography was then obtained. There was no evidence of tear of the Achilles or anteromedially located plantaris (Figure 2). The study demonstrated reproducible plantaris tendon subluxation from the medial margin of the Achilles tendon to a more posterior position (Figure 3, A and B) (Supplemental Video 2, http://links.lww.com/JG9/A122).

Despite physical therapy and activity modification, her pain persisted. She reported daily aching pain (level 4 out of 10) worse with weight bearing and physical activity. She attempted to return to dance but reported the pain limited her ability to dance. Given her persistent symptoms, she underwent a plantaris tenotomy.

### Surgical Treatment

**Figure 4:**
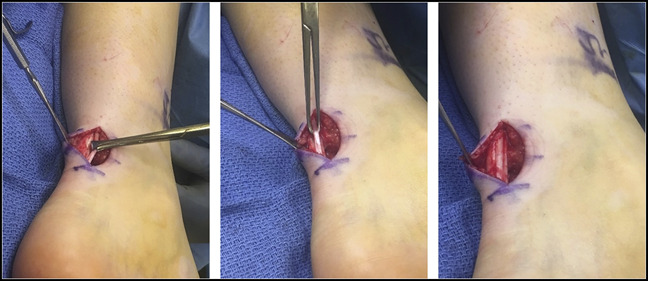
Intraoperative images showing the open left plantaris tenotomy. The plantaris tendon was identified medial and adjacent to the Achilles tendon. The tendon was dissected free from the Achilles and is shown being held by an Allis clamp before tenotomy.

The site of snapping was marked preoperatively, provoked by squatting. This was again identified just medial to the Achilles tendon. An approximately 3-cm incision was made overlying the snapping tendon, with careful dissection through the soft tissue down to the plantaris tendon. On inspection, no vessels or other structures were identified as possible causes of snapping. The tendon was normal-appearing in caliber without tear, hypertrophy, or associated tenosynovitis. The plantaris tendon closely abutted the medial margin of the Achilles tendon, and there were no vincula interconnecting the two tendons. The Achilles was also noted to be without apparent degeneration or injury (Figure 4). At this point, a tenotomy was done by transecting the plantaris tendon at the proximal and distal extent of the incision. The ankle was plantarflexed and tension was pulled on the tendon to do the tenotomy, thus removing a section of tendon. Copious irrigation was done followed by layered closure and splinting.

Postoperative care involved restricted weight bearing in a short-leg splint for 2 weeks. Sutures were removed at 2 weeks, and she progressed weight bearing in a tall controlled ankle motion boot. By 8 weeks postoperatively, she had resumed near-normal activity level, including returning to dance. Physical therapy was done postoperatively to improve strength and range of motion. She was seen 4.5 months postoperatively and reported 90% improvement, with mild residual pain around the Achilles when dancing nearly daily.

Patient-reported outcome measures demonstrated excellent clinical improvement. She reported a preoperative Foot and Ankle Ability Measure (FAAM) Activities of Daily Living score of 89.28. Twenty-three months postoperatively, she reported a full recovery with no residual pain, with FAAM Activities of Daily Living, FAAM Sports Subscale, and Visual Analog Scale scores of 100, 100, and 0, respectively.

## Discussion

The plantaris is a small accessory ankle plantar flexor and knee flexor adjacent to the triceps surae.^7,11^ The plantaris originates at the lateral supracondylar line of the femur, medial and superior to the lateral head of the gastrocnemius and then has a small fusiform muscle belly that extends 7 to 10 cm in length.^12,13^ The muscle travels slightly deep relative to the lateral head of the gastrocnemius, and the myotendinous junction typically is at the same level as the origin of the soleus.^12,14^ The tendon, often referred to as the “freshmen's nerve” because of its long and thin structure that can be mistaken for a nerve, travels medially within the avascular area between the gastrocnemius and underlying soleus, before inserting medially into the calcaneus or Achilles tendon.^11,15,16^ Anatomical studies have categorized this insertion into as many as nine types, with a majority inserting into the calcaneus.^17,18,19,20^ Van Strekenburg et al^20^ found that among paired legs, only 14% had identical insertion sites. The plantaris is innervated by the tibial nerve and perfused by the superior lateral genicular, popliteus, and lateral sural arteries proximally. Distally, it is perfused by the posterior tibial artery and is surrounded by the same peritendinous tissues as the Achilles tendon.^13,16^

The plantaris is a weak flexor, contributing a mean of 0.7% of total ankle plantar flexion—considerably less than the adjacent soleus (29.9%), medial head gastrocnemius (13.7%), and lateral head gastrocnemius (5.5%).^21^ The plantaris, which has a high density of muscle spindles, is believed to provide proprioception for the surrounding plantar flexors. However, because of its minor role in plantar flexion strength, it is a common tendon graft source.^11^ Cadaveric studies have described its absence in up to 19% of lower extremities, with upward of 13% of individuals demonstrating bilateral absence.^22,23^

Plantaris ruptures occur relatively frequently, often during jumping or running and because of eccentric loading of the ankle with the knee extended.^24^ Proximal strains of the muscle belly have been associated with anterior cruciate ligament injury.^14^ Ruptures at the myotendinous junction are the most frequent site of tears and can occur with injury to the medial head of the gastrocnemius.^14,16^ Plantaris ruptures may also occur in association with Achilles rupture.^25^ Although less common, distal insertional plantaris ruptures have been described.^26^ Plantaris ruptures may be mistaken for other pathology, including thrombophlebitis, deep vein thrombosis, Achilles tendon tear, or a ruptured Baker's cyst.^7,25^ Ruptures are treated nonsurgically, often with immobilization followed by gentle rehabilitation.

The plantaris has also been indicated in mid-substance Achilles tendinopathy.^8,20,27^ Although the exact cause is not completely understood, different mechanisms have been proposed. Some speculate that individuals with close anatomic proximity of the two tendons may experience compression or shearing, potentially provoking Achilles pathology.^16,28,29^ Smith et al^30^ found that baseline multidirectional differential motion exists between the plantaris and the Achilles, and thus, chronic stress between the tendons may result in Achilles pathology. Spang et al^16^ proposed that isolated plantaris tendinopathy can contribute to Achilles pathology. Countering the proposed compressive mechanism, a recent retrospective MRI study of mid-substance Achilles tendinopathies found a low correlation between plantaris and Achilles tendinopathy locations. Despite this dissonance, coexisting tendinopathies were found in 10% of the examined cases.^31^ When conservative treatments for mid-substance Achilles tendinopathy with associated plantaris pathology fail, plantaris excision with or without Achilles tenosynovectomy has demonstrated good surgical outcomes for both recreational and high-level athletes.^32-34^

Our review of the English literature revealed only two reported cases of a snapping plantaris tendon: one after a skateboarding accident and the other in an amateur runner.^9,10^ In the skateboarding accident, the authors hypothesized that the plantaris may have subluxated from its shared Achilles paratenon layer.^9^ Because our patient denied a specific traumatic event, it is difficult to determine a clear cause. We did not identify an anatomic abnormality intraoperatively that might explain why she had developed snapping. We speculate that the repetitive stress of dancing somehow contributed to its development, although concede that we cannot be certain. The snapping plantaris was diagnosed clinically and by dynamic ultrasonography. This pathology resulted in pain refractory to physical therapy, yet resolvable by plantaris tenotomy.

## Conclusion

After failed nonsurgical treatment of a snapping plantaris tendon with physical therapy, an open plantaris tenotomy enabled a 15-year-old female competitive dancer to return to dancing without pain. This case demonstrates a successful course of treatment of this rare pathology for a competitive dancer.

## Supplementary Material

SUPPLEMENTARY MATERIAL
